# β-Hydroxyphosphocarnitine modifies fibrosis, steatosis and improves liver function in non-alcoholic steatohepatitis induced in rats

**DOI:** 10.1186/s40360-022-00613-2

**Published:** 2022-09-29

**Authors:** Janet Sánchez-Quevedo, Emmanuel Ocampo-Rodríguez, Elizabeth Alvarez-Ayala, Anahí Rodríguez-López, Miguel Angel Duarte-Vázquez, Jorge Luis Rosado, Lourdes Rodríguez-Fragoso

**Affiliations:** 1 Facultad de Farmacia, Universidad Autónoma del Estado de Morelos, Av. Universidad 1001, Col. Chamilpa Cuernavaca, Morelos, Mexico; 2 Nucitec S.A. de C.V., Santiago de Querétaro, Mexico

**Keywords:** Non-alcoholic steatohepatitis, Fibrosis, Steatosis, Inflammation, β-hydroxyphosphocarnitine

## Abstract

**Background:**

Non-alcoholic steatohepatitis (NASH) is a chronic disease characterized by inflammation, steatosis, and liver fibrosis. The liver is particularly affected by alterations in lipid metabolism. Our aim was to evaluate the effect of β-hydroxyphosphocarnitine (β-HPC) on NASH induced in rats.

**Methods:**

NASH was produced via the ad libitum daily chronic administration of a fructose solution (400 kcal) for 9 weeks, an oral dose of fat solution (16 kcal) for 7 weeks and a subcutaneous injection of CCl_4_ (30%) two times a week for 2 weeks to Wistar rats. To evaluate the effect of β-HPC, a dose of 100 mg/kg was administered perorally for 4 weeks and its biochemical and hepatic effects on rats with NASH were analyzed. Serum levels of glucose, triglycerides, cholesterol, and liver enzymes were quantified. Histological changes were evaluated on slices stained with H&E, trichromic and PAS. Glycogen content was measured in liver samples. α-SMA and SREBP-1 immunopositive cells were identified in liver tissue.

**Results:**

NASH was characterized by elevated triglycerides, elevated liver damage enzymes, and the presence of necrosis, inflammation, steatosis, and fibrosis. Significant amounts of glycogen were found, along with α-SMA positive cells in fibrosis areas. The over-expression of SREBP-1 in cytoplasm and nuclei was evident. Animals with NASH treated with β-HPC showed a significant reduction in inflammation, necrosis, and glycogen content in the liver. A reduction in α-SMA and SREBP-1 immunopositive cells correlated with a significant reduction in the degree of fibrosis and steatosis found in liver tissue. β-HPC reduced the levels of ALP and GGT, and significantly reduced triglyceride levels. Animals treated with β-HPC did not show any alterations in liver enzyme function.

**Conclusions:**

Our research shows that β-HPC can improve liver function and morphology in the case of NASH induced in rats, suggesting β-HPC could be potentially used in the treatment of NASH.

## Background

Technological and industrial development have led to an increase in sedentary lifestyles that, along with the excessive consumption of processed foods, have led to the development of a wide variety of emerging metabolic diseases, including metabolic syndrome and chronic liver diseases [1–3]. Non-alcoholic steatohepatitis (NASH) is a chronic and progressive liver disease that begins as steatohepatitis and, due to various factors, can lead to the development of fibrosis, cirrhosis, and liver cancer [4]. The liver is particularly affected by alterations in lipid metabolism due to the essential role it plays in energy homeostasis [5]. Various studies attribute the accumulation of fat in the liver to two main sources: diet and de novo lipogenesis (synthesis of fatty acids from consumed excess carbohydrates) [6]. The presence of steatosis and a chronic inflammatory state lead to a distortion of the liver parenchyma with progressive deterioration of liver function [7] and development of fibrosis [8].

We are currently in urgent need of therapies that improve histological abnormalities and prevent the progression of liver fibrosis [9]. Due to the lack of adequate therapy, NASH has become the focus of important research [10]. Current available treatments are pioglitazone, vitamin E, GLP (glucagon-1-like peptide) agonists, and pentoxifylline [11]. However, these treatments have little effect when seeking to improve the different comorbidities associated with NASH. This leads to the administering of different drugs depending on the need of each NASH patient [12]. Said drugs can produce severe side effects such as weight gain, decreased bone density, prostate cancer and cerebrovascular disease, which means they cannot be used for prolonged periods [13]. β-hydroxyphosphocarnitine (β-HPC) is an L-carnitine analog that has been shown to effectively reduce insulin levels and serum levels of glucose, triglycerides and cholesterol in liver and serum from obese Zucker fa/fa rats as well in rats turned insulin resistant by a high-fructose diet [14, 15]. Previous studies regarding its toxicity have demonstrated that β-HPC has an excellent safety margin in preclinical studies [16]. Pharmacokinetic studies of β-HPC showed higher absorption compared to its precursor, L-carnitine [17]. Given the positive effect of β-HPC in metabolic alterations, it is possible it could be effective for NASH treatment. Therefore, the goal of the present study was to evaluate the pharmacological effect of β-HPC on NASH induced in rats.

## Methods

### Test material and animals

β-HPC (β-Hydroxyphosphocarnitine) was obtained from NUCITEC S.A. de C.V. (Qro., Mexico) and dissolved in sterile deionized water before experimental use. Adult male Wistar rats weighing 120 g (Envigo Laboratories Inc. Mexico) were used. The animals were housed in controlled ambient temperature and humidity, maintained with a standard diet (Standard Purina Chow Diet, Mexico) and had water ad libitum. The animals were treated in accordance with the Guide for the Care and Use for Laboratory Animals [18].

### Non-alcoholic steatohepatitis (NASH) model induced in rats

The NASH model was developed using the method already reported in the literature [19], with some modifications. Briefly, fructose solution (10%, 400 kcal), a sutured fat solution (Lauric acid 22.3, myristic acid 8.4, palmitic acid 4.1, caprylic acid 3.75, capric acid 3, stearic acid 1.4, and caproid acid 4.3 g/mL, (16 kcal) and carbon tetrachloride (CCl_4_, 30%) were administered to rats as follows: the animals were allowed to ingest a fructose solution ad libitum in place of drinking water, from the first day until the end of the study (9 weeks); during the second week, animals started to receive a 2 mL daily oral dose of sutured fat solution using an intragastric tube, and this lasted until the end of the study; during the third week, rats received a subcutaneous injection CCl_4_ (0.2 mL/100 g body weight) diluted in mineral oil two times a week for 2 weeks. Once these two weeks were over, NASH was considered established and was verified histologically. After this period, the animals continued to receive a single dose per week of CCl_4_ until the 9 weeks were completed. In addition to this, all animals were maintained with a standard diet for 9 weeks.

### Pharmacological treatments

The rats were grouped into 4 groups of 5 rats each: (1) Control, animals with no treatment; (2) β-HPC, (3) NASH, and (4) NASH + β-HPC. The β-HPC was dissolved in water and administered orally at a dose of 100 mg/Kg through a cannula. The β-HPC dose was chosen in accordance with previous results of studies carried out in animals treated with this drug [14, 15]. The β-HPC treatment started on week 5, once NASH had been established, and was administered for the following 4 weeks. The animals continued with their standard diet and only drank the fructose solution instead of drinking water.

After treatment, the rats were deprived of food and received only water for 12 h, after which animals were euthanized with pentobarbital sodium (150 mg/Kg) i.p. The blood samples were centrifuged to separate the serum. Fragments of tissue were fixed in 4% formaldehyde, then embedded in paraffin to perform histological cuts. The serum was kept under -70 °C until analysis. These experimental procedures were performed in accordance with the guidelines for animal experimentation of the Autonomous University of the State of Morelos (Mexico), Welfare Act Public Laws of Morelos State (Mexico) and reported according to the ARRIVE guidelines [20].

### Histopathological analysis of liver

Histological sections of liver stained with Hematoxylin & Eosin, Masson's trichromic and Periodic acid Schiff (PAS) were made. Tissue sections and staining were performed by a pathologist (INP, S.S.) with one researcher (FF, UAEM) blinded with regards to the length storage. Overall interobserver difference was 5%. The tissue sections were observed under light microscopy and the degree of damage was evaluated based on the Metavir and Ishak Score criteria [21, 22], where: N0 corresponds to non-necrosis, N1 corresponds to mild necrosis (few areas), N2 corresponds to mild / moderate necrosis (most of the areas), N3 corresponds to moderate necrosis (continuous < 50%), and N4 corresponds to severe necrosis (continuous > 50%). For the evaluation of focal inflammation, I0 corresponds to no focus per field, I1 corresponds to one focus or less per field, I2 corresponds to two to four sources per field, I3 corresponds to 5 to 10 sources per field and I4 corresponds to more than 10 foci per observed field. For the evaluation of portal inflammation, PI0 corresponds to none, PI1 corresponds to mild (some or all portal areas), PI2 corresponds to moderate (some or all portal areas), PI3 corresponds to moderate / marked (all portal areas), PI4 corresponds to marked (all portal areas). For the evaluation of hepatocytes in balloon, B0 corresponds to none, B1 to few cells in balloon and B2 to many cells in balloon.

The degree of fibrosis was determined according to the Ishak criterion that classifies fibrosis in 7 stages; where stage 0 has very little fibrous tissue in the portal areas and central vein walls, stage 1 shows fibrous expansion of the portal spaces, which may maintain a rounded contour or develop short spike-shaped septa, first involving only a few portal spaces and eventually all portal spaces (stage 2). In stage 3, the fibrous septa extend to form bridges between adjacent vascular structures, both portal-to-portal and portal-to-central, progressing to numerous bridges or septa (stage 4). In stage 5 you can see the formation of parenchymal nodules completely surrounded by fibrosis, indicating early or incomplete cirrhosis; when the tissue is composed entirely of nodules it can be considered as cirrhosis (stage 6). PAS staining procedure was used to detect glycogen deposits in the liver. Liver cells with purple color showed an abundant presence of glycogen.

Serum was collected by centrifugation of the blood and was used for the quantification of glucose, triglycerides (TG) and cholesterol. The levels of glucose, triglycerides and cholesterol were determined by colorimetric methods (Glucose PAP SL, Triglycerides SL and Cholesterol PAP SL, ELITech, Mexico). Serum enzyme activity of alanine aminotransferase (ALT), aspartate aminotransferase (AST), alkaline phosphatase (ALP) and gamma glutamyl transferase (GGT) were quantified by colorimetry using a commercial reagent kit (ELITech, Mexico).

### Glycogen quantification

For liver glycogen quantification, 250 mg of liver tissue from each rat were weighed. Liver tissue was digested with 3% sodium hydroxide in a water bath for 20 min. Two dilutions were made with the digested tissue. Subsequently, 1 mL of the digested tissue solution was taken and 1.5 mL of anthrone solution (0.2% anthrone/95% sulfuric acid (H_2_SO_4_)) were added and placed again in a water bath for 10 min. The samples were read in a Victor 3 Perkin Elmer spectrophotometer (MTX Lab Systems, Inc., VA, and USA) at an absorbance of 550 nm.

### Immunohistochemical detection for activated hepatic stellate cells and SREBP-1 positive cells

The identification of hepatic stellate cells (HSC) in liver slices was made by immunofluorescence using an alpha smooth muscle actin (α-SMA) antibody. Analysis was developed in 2 mm-tick slices; tissue sections were deparaffinized and rehydrated, and antigen retrieval was performed with a target retrieval citrate solution (Dako Corporation, CA, and USA). Endogenous peroxidase was inactivated with 0.9% H_2_O_2_ (hydrogen peroxide), and washes were performed with distilled water. Then slices were allowed to stand for 5 min in phosphate-buffered saline (PBS). Tissue sections were incubated for 60 min with monoclonal anti-α-SMA (sc-32251, Santa Cruz Biotechnology, CA, USA) 1:50 dilution. Following incubation with the primary antibody, sections were incubated with FITC conjugated rabbit anti-mouse 1:200 dilution for 10 min (Jackson Laboratories Inc Immuno Research, PA, USA). Images were taken (40X) using an Olympus camera (America Inc, Pennsylvania, US) and the ImagePro (Media cybernetics, Inc.) program. The identification of Sterol Regulatory Element Binding Protein -1(SREBP-1) positive cells was carried via a primary antibody against SREBP-1 (sc365513, Santa Cruz Biotechnology, CA, USA) in 1:40 dilution for 40 min. After time was using a mouse/rabbit polydetector plus DAB HRP brown detection system and following the supplier´s instructions (Bio SB Mexico, Mex). Our positive control tissue was human salivary gland tissue showing cytoplasmic and nuclear brown staining.

### Statistical analysis

The data were presented as the mean + standard deviation (SD). The statistical analysis was performed using SPSS 17 Real Stat software (Armonk, NY, USA). The statistical differences between groups were determined by ANOVA, followed by Tukey’s test. Values were considered as statistically significant at *p* < 0.05.

## Results

**Fig. 1:**
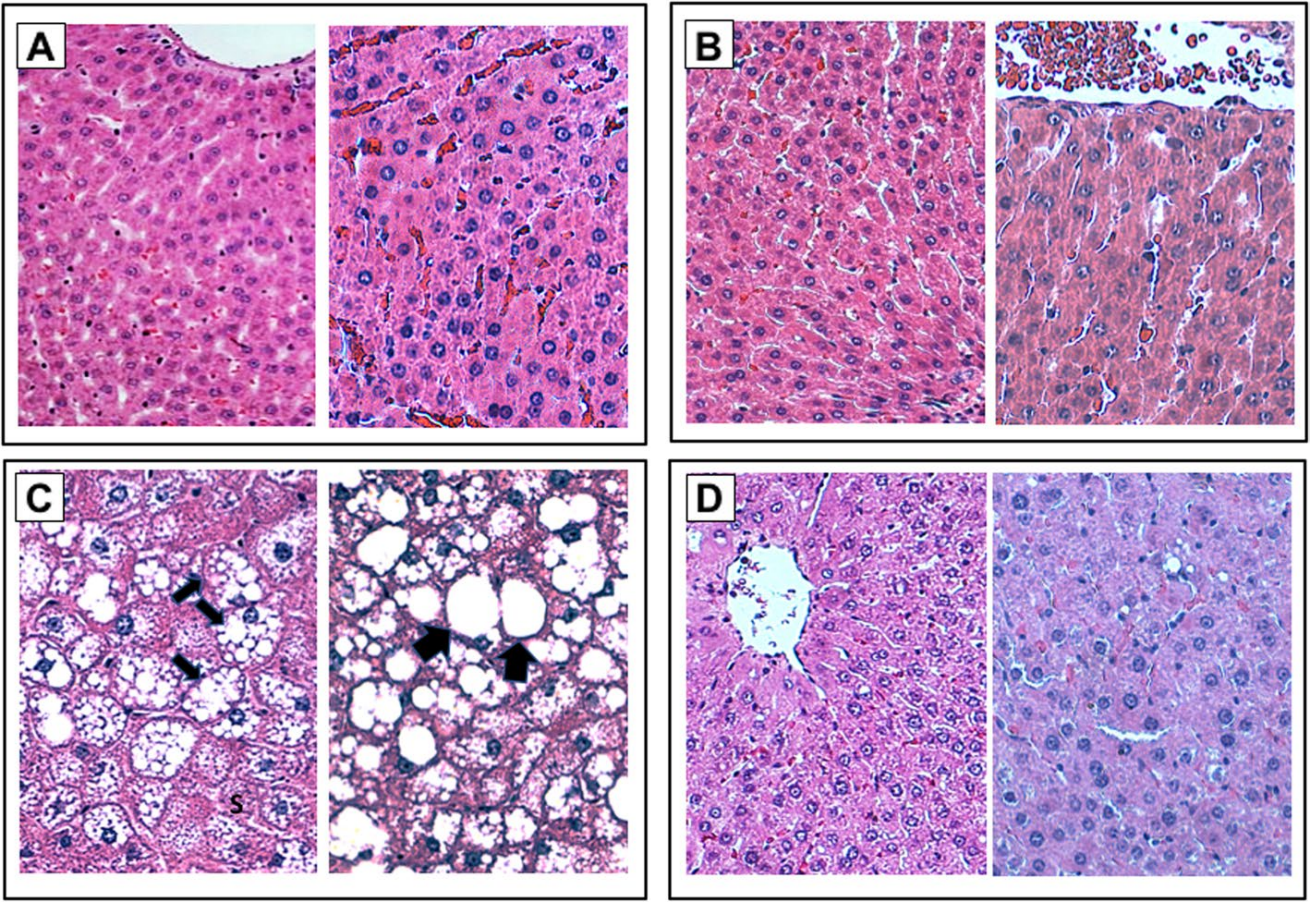
Effect of β-HPC on liver architecture in rats with NASH. Histopathological analysis of liver sections from: **A** Control group; **B** β-HPC group; **C** Non-treated NASH group, showing distortion of hepatic architecture with steatosis (S), and bands of fibrosis (F); **D** NASH group treated with 100 mg/Kg β-HPC, showing an improvement in liver structure as well as reduced steatosis and fibrosis. Black arrows show presence of microvesicular and macrovesicular steatosis.The liver sections were stained with H&E stain. Magnification 20X and 40X

Histological sections from male Wistar rats were stained with H&E, Masson's trichromic and PAS stain to identify histopathological changes, fibrosis and accumulation of glycogen. The histological sections of the control group and β-HPC showed normal morphology of the liver parenchyma (Fig. 1A, and B). Histological sections of the NASH rats showed alterations and damage to the parenchyma, including balloon hepatocytes, necrosis, microvesicular steatosis, and liver cord dissociation (Fig. 1C). In the NASH group treated with β-HPC there was a reduction in balloon hepatocytes, necrosis, inflammation, steatosis and fibrosis, and the improvement in the structure and morphology of the tissue was evident (Fig. 1D). We performed a classification by degree of damage of the histological sections (Table 1). 100% of the rats in the control group were classified as necrosis grade 0; 60% of the rats in the NASH group treated with β-HPC as grade 3 and, among the animals in the non-treatment NASH group, 80% of the rats were in grade 3. The reduction in balloon hepatocytes was evident in the NASH group treated with β-HPC as compared to the non-treated NASH group, which reached a higher grade (2). The analysis showed a reduction of focal inflammation in the NASH + β-HPC group, with 60% of the rats in grade 3 compared to the NASH group that had 100% of the rats in grade 3.

**Table 1:** Effect of β-HPC on histopathological changes of the liver

***Groups***	***Necrosis***								***Ballooned hepatocytes***					
	**Score (# of animals)**								**Score (# of animals)**					
	**0**	**1**		**2**	**3**		**4**		**0**		**1**			**2**
**Control**	5	0		0	0		0		5		0			0
**β-HPC**	5	0		0	0		0		5		0			0
**NASH**	0	0		1	4		0		0		0			5
**NASH + β-HPC**	0	0		2	3		0		5		0			0
**Groups**	**Portal inflammation score (# of animals)**								**Focal inflammation score (# of animals)**					
	**0**	**1**		**2**	**3**		**4**		**0**	**1**	**2**		**3**	**4**
**Control**	5	0		0	0		0		5	0	0		0	0
**β-HPC**	5	0		0	0		0		5	0	0		0	0
**NASH**	0	5		0	0		0		0	0	0		5	0
**NASH + β-HFC**	0	5		0	0		0		0	0	2		3	0
	**Grading and stages of fibrosis (# of animals)**													
	**0**		**1**			**2**		**3**		**4**		**5**		**6**
**Control**	5		0			0		0		0		0		0
**β-HPC**	5		0			0		0		0		0		0
**NASH**	0		0			5		0		0		0		0
**NASH + β-HPC**	0		5			0		0		0		0		0

Values represents the number of animals per group, classified by degree of damage. The analysis was based on the Metavir and Ishak criteria

**Fig. 2:**
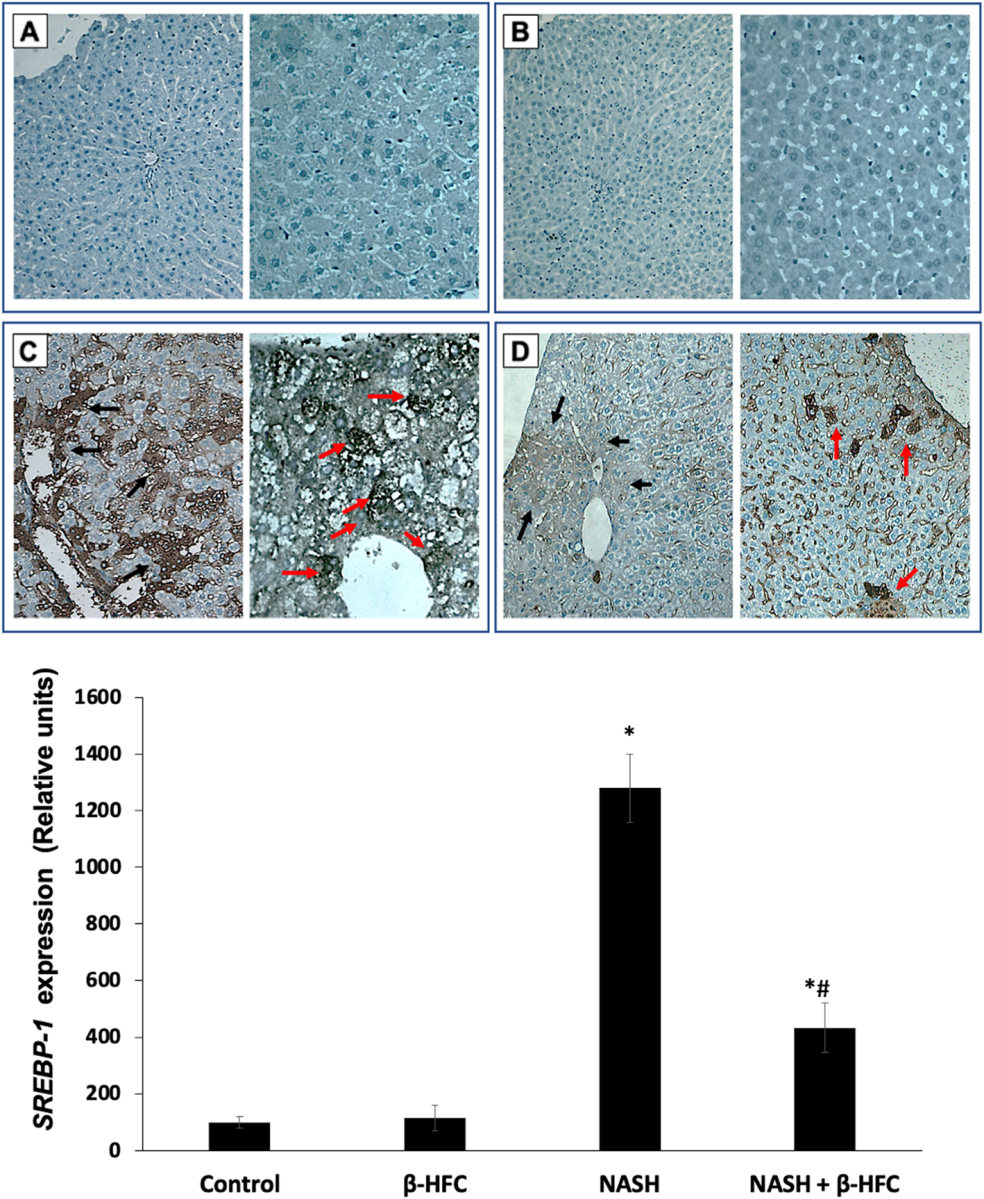
Effect of β-HPC on *SREBP-1 expression* in rats with NASH. The expression of *SREBP-1* was determined by immunohistochemistry. Positive reactions can be seen in brown. **A** and **B** Livers from control and β-HPC groups respectively: staining reveals scattered isolated positive cells throughout the parenchyma. **C** Non-treated NASH group: *SREBP-1* localization in liver with steatosis and fibrosis: many immunopositive cells were observed throughout the parenchyma. Black arrow indicates positive cytoplasm stain. Red arrow indicates positive nuclei stain. **D** NASH group treated with 100 mg/Kg β-HPC: showing a reduced amount of immunopositive cells: there were occasional and faint immune positive hepatocytes through the parenchyma. Magnification of 40X

An over-expression of SREBP-1 protein was present in wide areas inside the cytoplasm of liver cells in rats with NASH. The presence of SREBP-1 in the nuclei of some cells indicated the activation of this transcriptional factor. The expression of SREBP-1 correlated with the presence of liver steatosis. A significant reduction in the degree of steatosis correlated with a minimal expression of SREBP-1 in the NASH group treated with β-HPC (Fig. 2).

**Fig. 3:**
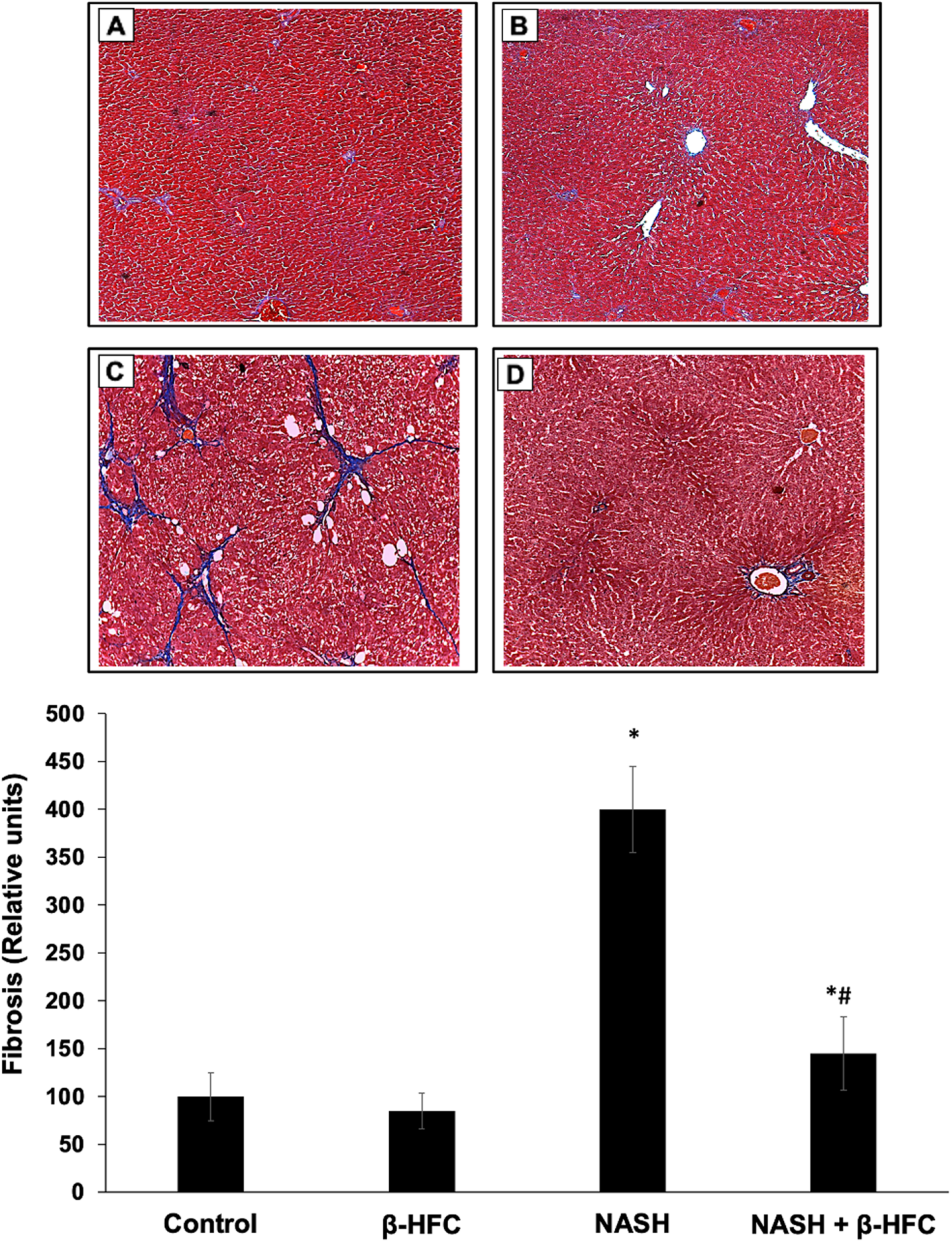
Effect of β-HPC on liver fibrosis in rats with NASH. **A** Control group: normal lobular architecture and normal distribution of collagen are shown. **B** β-HPC group: normal distribution of collagen around the vessels is shown. **C** Non-treated NASH group: extensive collagen deposition in parenchyma and formation of bridges of collagen in liver tissue were observed. **D** NASH group treated with 100 mg/Kg β-HPC: showing a reduced deposition of collagen in parenchyma and reduction of the collagen bridge. Representative histological sections of the liver from each group, Masson's trichromic stain, and white arrows showing the presence of collagen (Magnification 4X)

**Fig. 4:**
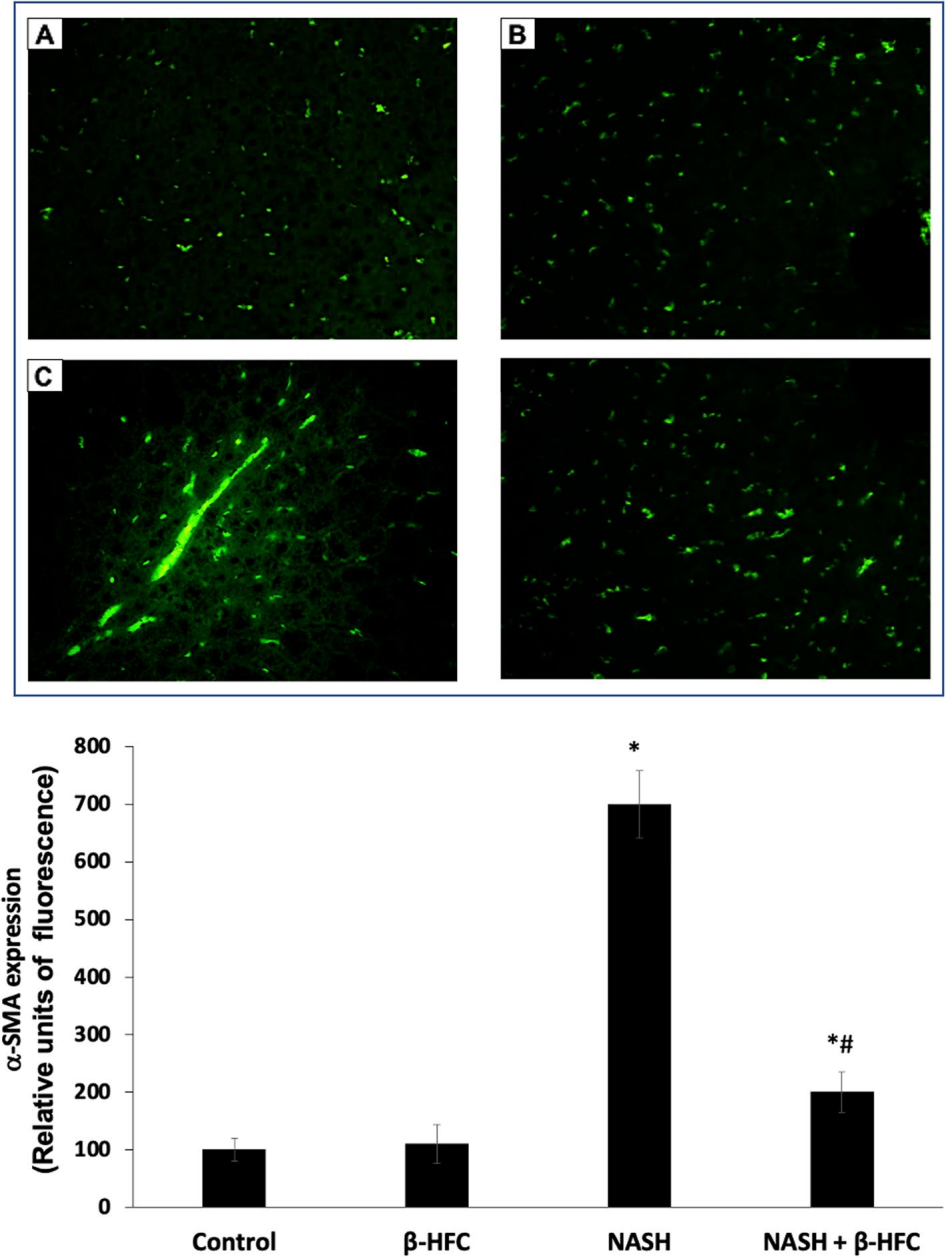
Effect of β-HPC on α-SMA expression and localization in rats with NASH. The localization of α-SMA in liver slices was determined using immunofluorescence. Positive cells show green in color. **A** Control group. **B** β-HPC group: isolated α-SMA cells are seen throughout the parenchyma. **C** Non-treated NASH group: localization of α-SMA in liver tissue. Immunoreactive cells were observed in the bridges of collagen, indicating activated HSC. **D** NASH group treated with 100 mg/Kg -1–1: reduced amount of immunoreactive cells, α-SMA cells were distributed throughout the parenchyma and no presence of bridges was detected. Magnification of 40X

The effect of β-HPC on liver fibrosis was observed in histological sections stained with Masson's trichromic, which shows the collagen fibers in blue staining. Collagen fibers can be found around the central veins in moderate quantities as observed in the histological sections of the control and β-HPC groups (Fig. 3A and B). When liver damage persists, there is a thickening of the collagen fibers around the central veins and the formation of small collagen fiber bridges that invade the liver tissue (Fig. 3C). The degree of fibrosis in the NASH group treated with β-HPC showed reduced fibrosis development when compared to the non-treated NASH group (Fig. 3D). α-SMA expression was analyzed as a marker of activated HSC in liver slices from all groups (Fig. 4). α-SMA expression in the tissue from animals with NASH showed the presence of α-SMA positive cells in fibrosis areas, indicating the presence of activated HSC (Fig. 4C). The α-SMA expression in liver slices from animals treated with β-HPC showed a smaller number of positive cells within the parenchyma (Fig. 4D), which correlated with reduced presence of fibrosis.

**Fig. 5:**
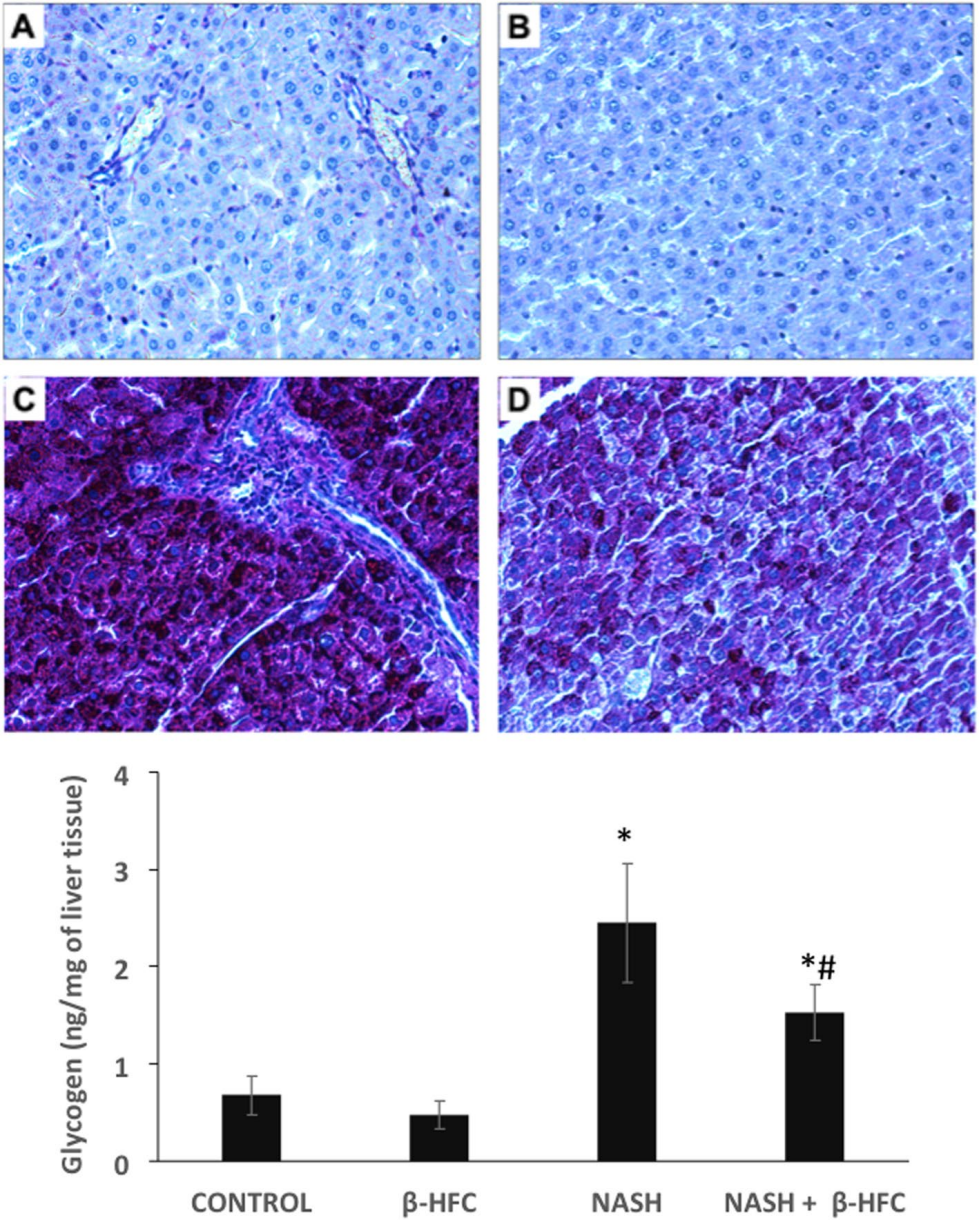
Effect of β-HPC on liver glycogen content in rats with NASH. **A** Liver glycogen in the control group: PAS-positive areas mainly in the cytoplasm of hepatocytes. **B** NASH groups. **C** Liver glycogen in the non-treated NASH group: PAS-positive cells were significantly increased throughout liver parenchyma. **D** Liver glycogen in NASH group treated with β-HPC: PAS-positive cells were observed in liver parenchyma. Liver glycogen was demonstrated by Periodic acid–Schiff (PAS) staining, dark areas indicate presence of glycogen (magnification 40X). Each bar represents the mean ± SD; experiments were performed in duplicate. ^*^p < 0.05 compared to control group, ^#^*p* < 0.05 compared to NASH group without treatment. All groups consisted of 5 animals

PAS staining was used to identify glycogen synthesis and functional hepatocytes. Animals with NASH showed a great amount of glycogen in liver parenchymal cells. However, animals with NASH and treated with β-HPC showed a very small presence of glycogen. The quantification of hepatic glycogen showed an increase of 3.6-fold when compared with the control group (Fig. 5). Animals with NASH receiving β-HPC showed a significant reduction (40%) in glycogen content (*p* < 0.05).

The quantification of liver enzyme levels is commonly used to diagnose liver damage. The enzymes ALT and AST are released into the blood flow when cell and mitochondrial membranes are damaged. Cell damage was evident in animals with NASH: their ALT levels were 3.3-fold those of the control group (*p* < 0.05). Animals with NASH and treated with β-HPC did not show any modification in ALT levels. In the case of AST, the values in the NASH group increased 3.2-fold when compared with the control group (*p* < 0.05). Rats with NASH and treated with β-HPC had a 23% reduction in AST levels when compared to the NASH group, but this was not statistically significant (Table 2).

**Table 2:** Liver function test

**Enzyme**	**Control**	**β-HPC**	**NASH**	**N + β-HPC**
AST (U/L)	30.7 ± 2.2	31.7 ± 12.1	101.3 ± 17.3*	77.7 ± 11.9
ALT (U/L)	25.9 ± 8.9	33 ± 9.9	85.7 ± 10.0*	74.3 ± 11.6
ALP (U/L)	127.3 ± 14.8	125.2 ± 29.3	556.4 ± 48.0*	327.4 ± 31.7 *#
GGT (U/L)	0.90 ± 0.1	0.81 ± 0.1	3.21 ± 0.3*	1.00 ± 0.5**#**
Glucose (U/L)	123.2 ± 50.6	102 ± 33.3	134.3 ± 19.1	118.4 ± 31.7
Triglycerides (mg/dL)	63.7 ± 12.65	38.5 ± 8.74**#**	255.1 ± 28.49*	48.4 ± 13.40**#**
Cholesterol (mg/dL)	57.9 ± 8.66	47.5 ± 8.85	42.3 ± 9.46	37.5 ± 7.11

Mean ± SD; experiments were performed in duplicate

^*^p < 0.05 compared to control group, #*p* < 0.05 compared to NASH group without treatment. All groups consisted of 5 animals

The ALP and GGT enzymes are located in the epithelial area of the bile duct and their elevation suggests reduction or obstruction of bile production and the presence of cholestasis. The rats in the NASH group had a 4.3-fold increase in ALP levels as compared with the control group (*p* < 0.05). However, animals with NASH and treated with β-HPC showed a 41% reduction in ALP levels compared with rats in the non-treated NASH group (*p* < 0.05). In the case of GGT, the rats with NASH had a 3.5-fold increase compared to the control group (*p* < 0.5). The NASH group treated with β-HPC showed a 69% reduction compared to the non-treated NASH group (*p* < 0.05). Animals treated with β-HPC did not show any alterations in liver enzyme function.

During development of NASH, there are many biochemical alterations that include changes in glucose, triglyceride, and cholesterol levels. The rats with NASH showed elevated triglyceride levels, fourfold compared to the control group (*p* < 0.05). Interestingly, animals with NASH but treated with β-HPC showed an important reduction in triglycerides (80%) when compared to the non-treated NASH group (*p* < 0.05) (Table 2). Interestingly, animals treated only with β-HPC showed a significant reduction in triglyceride levels. The glucose and cholesterol levels in rats with NASH did not significantly differ from those in the control group. Animals treated with β-HPC did not show any biochemical alterations in this regard.

## Discussion

Our current human lifestyle leads to many chronic ailments [23]. More specifically, the literature reports that fats and fructose from fast foods and processed beverages are responsible for the development of diseases such as NASH [24–29]. NASH is a pathology associated with a set of metabolic alterations that make its treatment difficult, and there is no current specific pharmacological treatment [30, 31].

L-carnitine is an essential amino acid in the transportation of fatty acids to the mitochondrial matrix and is used in the treatment of metabolic disorders, mainly obesity [32]. It plays a relevant role in the pathology of NAFLD, counteracting the inflammatory process of the liver by upregulating the Peroxisome Proliferator Activator Receptor-γ (PPAR-γ) [33]. β-HPC is an L-carnitine analog that has been shown to have greater absorption than its precursor while maintaining the pharmacological properties [14, 17]. This study evaluated the effect of β-HPC in a rat model where NASH was induced by the administration of a fructose solution (400 kcal), a sutured fat solution (16 kcal) and CCl_4_ (30%) for 9 weeks. This and other NASH models mimic the factors that lead to the development of biochemical alterations and changes in liver morphology in humans [19, 34, 35].

The results showed that rats with NASH had increased serum triglyceride (TG) levels, as well as NASH-characteristic liver structural changes (inflammation, necrosis, steatosis, and fibrosis), which was demonstrated by histological analysis. We know that liver fibrosis in NASH develops through signaling pathways that are activated by crosstalk between hepatocytes, HSC and immune cells, all of which is associated with hepatocellular injury and functional changes of the organ [36]. The rats with NASH that were also treated with β-HPC showed less thickness and no bridging of collagen fibers, which indicates a lower degree of fibrosis. It has been suggested that the HSC activation and transcriptional plasticity that occur during NASH development play an important role in the fibrogenesis associated with this disease [37, 38]. The present study clearly showed immunepositive α-SMA cells which indicated HSC activation in animals with NASH, which in turn correlated with the presence of fibrosis in liver tissue. The reduction of immunepositive α-SMA cells in animals with NASH and treated with β-HPC suggests this might interfere with pathways associated with the activation of HSC.

The SREBP are a family of transcriptional factors involved in lipid homeostasis (cholesterol, fatty acid and triglycerides) [38]. We now know that the activation of SREBP-1 leads to fatty acid synthesis via the activation of genes related with lipogenic pathways [39]. Hepatic triglyceride accumulation is due to an endoplasmic reticulum stress-induced by SREBP-1. It has been shown that fructose overingestion stimulates SREBP-1 expression, leading to hepatic lipid accumulation [40, 41]. The increase of SREBP-1 expression in animals with NASH might explain the increase in triglycerides and the presence of abundant fat vacuoles in the hepatic parenchymal. β-HPC led to a reduction in SREBP-1 expression, as well as in the number and size of fat vacuoles and also triglyceride levels in all animals with NASH, suggesting that β-HPC might modulate SREBP-1 expression and activation.

L-carnitine may play a key role in maintaining liver function through its effect on lipid metabolism. It has been previously reported that L-carnitine improves liver tissue alterations as well as the lipid profile in NASH because it reduces the levels of anti-inflammatory proteins [42]. It has also been reported that L-carnitine stimulates lipolysis-activity and increases the expression of genes involved in beta-oxidation, leading to the stimulation of different pathways associated with fat storage [43]. It is therefore possible that β-HPC could be producing the same effect.

The improvement in the liver function of rats with NASH treated with β-HPC was corroborated by liver enzyme levels commonly used as indicators of liver damage. The increase in ALP and GGT is associated with obstruction or decreased production of bile flow by hepatocytes, which can be caused by the extension and compression of the bile ducts due to fibrosis; therefore, the presence of fibrosis is also associated with an increase in these enzymes [44]. Lower levels of the GGT and ALP enzymes showed that β-HPC treatment had a positive effect on liver function in rats with NASH. Although ALT and AST enzymes also increased in animals with NASH, they tended to decrease in β-HPC-treated animals, suggesting that, in addition to fibrosis and steatosis, there was also a reduction in inflammation and necrosis, and this was reflected in an improvement of the functionality of the liver. Several studies have shown that L-carnitine is not only capable of reducing hepatic steatosis but also normalizes alterations in liver enzymes in patients with NAFLD [45].

## Conclusion

Since NASH is a chronic disease, most clinical patients require prolonged treatment for observable improvement. As already mentioned, the drugs used to treat NASH produce side effects and cannot be used for long periods of time, which makes them inappropriate. Alternatives are needed. Previous studies show β-HPC has a high safety margin [16], so it could be employed for long periods of time. While it was not possible to reduce all functional and structural NASH alterations of the liver in animals treated with β-HPC for 4 weeks, this study suggests that, if β-HPC is administered for a prolonged period, the improvement in liver architecture and its consequent functionality could increase. Therefore, β-HPC has the potential to be employed in NASH treatment.

## Data Availability

The datasets used and/or analyzed during the current study are available from the corresponding author on reasonable request.

## Abbreviations

NASH: Non-alcoholic steatohepatitis
GLP: Glucagon-1-like peptide
β-HPC: β-Hydroxyphosphocarnitine
CCl_4_: Carbon tetrachloride
PAS: Periodic acid Schiff
TG: Triglyceride
ALT: Alanine aminotransferase
AST: Aspartate aminotransferase
ALP: Alkaline phosphatase
GGT: Gamma glutamyl transferase
H_2_SO_4_: Sulfuric acid
HSC: Hepatic stellate cells
H_2_O_2_: Hydrogen peroxide
PBS: Phosphate-buffered saline
α-SMA: Alpha smooth muscle actin
SREBP-1: Sterol Regulatory Element Binding Protein-1
SD: Standard deviation
NAFLD: Non-alcoholic fatty liver disease
PPAR-γ: Peroxisome Proliferator Activator Receptor-γ
